# Microglial Inhibitory Mechanism of Coenzyme Q10 Against Aβ (1-42) Induced Cognitive Dysfunctions: Possible Behavioral, Biochemical, Cellular, and Histopathological Alterations

**DOI:** 10.3389/fphar.2015.00268

**Published:** 2015-11-09

**Authors:** Arti Singh, Anil Kumar

**Affiliations:** Pharmacology Division, University Institute of Pharmaceutical Sciences, UGC Centre of Advanced Study, Panjab UniversityChandigarh, India

**Keywords:** Alzheimer’s disease, Aβ (1-42), coenzyme Q10, minocycline, mitochondrial dysfunction, oxidative stress, neuroinflammation

## Abstract

**Rationale:** Alzheimer’s disease (AD) is a debilitating disease with complex pathophysiology. Amyloid beta (Aβ) (1-42) is a reliable model of AD that recapitulates many aspects of human AD.

**Objective:** The intent of the present study was to investigate the neuroprotective potential of Coenzyme Q10 (CoQ10) and its modulation by minocycline (microglial inhibitor) against Aβ (1-42) induced cognitive dysfunction in rats.

**Method:** Intrahippocampal (i.h.) Aβ (1-42) (1 μg/μl; 4μl/site) were administered followed by drug treatment with galantamine (2 mg/kg), CoQ10 (20 and 40 mg/kg), minocycline (50 and 100 mg/kg) and their combinations for a period of 21 days. Various neurobehavioral parameters followed by biochemical, acetylcholinesterase (AChE) level, proinflammatory markers (TNF-α), mitochondrial respiratory enzyme complexes (I-IV) and histopathological examinations were assessed.

**Results:** Aβ (1-42) administration significantly impaired cognitive performance in Morris water maze (MWM) performance test, causes oxidative stress, raised AChE level, caused neuroinflammation, mitochondrial dysfunction and histopathological alterations as compared to sham treatment. Treatment with CoQ10 (20 and 40 mg/kg) and minocycline (50 and 100 mg/kg) alone for 21 days significantly improved cognitive performance as evidenced by reduced transfer latency and increased time spent in target quadrant (TSTQ), reduced AChE activity, oxidative damage (reduced LPO, nitrite level and restored SOD, catalase and GHS levels), TNF-α level, restored mitochondrial respiratory enzyme complex (I, II, III, IV) activities and histopathological alterations as compared to Aβ (1-42) treated animals. Further, combinations of minocycline (50 and 100 mg/kg) with CoQ10 (20 and 40 mg/kg) significantly modulates the protective effect of CoQ10 dose dependently as compared to their effect alone.

**Conclusion:** The present study suggests that the neuroprotective effect of CoQ10 could be due to its microglia inhibitory mechanism along with its mitochondrial restoring and anti-oxidant properties.

## Introduction

Alzheimer’s disease (AD) is a slow growing neurodegenerative disorder, caused significant cognitive dysfunction worldwide (Hampel et al., 2011). It causes progressive loss of mental, behavioral, functional deficits and ability to learn (Braak and Del Tredici, 2012). Globally, it accounts for more than 36 million people worldwide suffering from this slow growing deadly disease (Anand et al., 2014; Association, 2014). The major pathological characteristics of AD include deposition of amyloid plaques (senile plaques) and neurofibrillary tangles (NFTs) (Zhong et al., 2009; Kumar and Singh, 2015). The amyloid plaques are primarily composed of amyloid beta (Aβ) (1-42) peptide. This peptide aggregates to form dimers and oligomers which are the principal toxic species that leads to neuronal death (Christensen et al., 2008).

Mitochondria are the major source of energy as well as both producers as well as targets of reactive oxygen species (ROSs), responsible for oxidative damage (Turrens, 2003; Medeiros et al., 2007; Reddy, 2007; Kumar and Dogra, 2009; Kumar and Singh, 2015). It has been reported that Aβ (1-42) administration causes an impairment of mitochondrial energy metabolism in different regions of the brain, which leads to progressive deterioration of mitochondrial functions, excessive free radical generation, eventually results DNA damage and cell death (Lenaz et al., 1998; Turrens, 2003; Reddy and Beal, 2005; Zeevalk et al., 2005; Reddy, 2007; Dumont and Beal, 2011; Federico et al., 2012; Kumar and Singh, 2015).

Research studies demonstrated that the Aβ (1-42) accumulation initiates a series of cellular cascades including glial cell (microglia and astrocytes) activation (Lenaz et al., 1998; Seabrook et al., 2006; Medeiros et al., 2007). The exact mechanism behind Aβ induced neuroinflammation hypothesis is still unknown (Seabrook et al., 2006). However, oxidative damage and mitochondrial dysfunction are considered to be responsible for shifting the cell signaling pathways toward proinflammatory and pro-apoptotic signals creating a link between oxidative stress and neuroinflammation (Rosales-Corral et al., 2010).

Since oxidative stress, mitochondrial dysfunction and neuroinflammation are all entailed in the pathogenesis of AD treatment with antioxidants along with mitochondrial restoring and anti-inflammatory property have been considered as one of the therapeutic strategy for the management of cognitive dysfunction including AD. Coenzyme Q10 (CoQ10) is a lipophilic, endogenous, vitamin-like antioxidant compound (Bentinger et al., 2010; Dumont et al., 2011). It has been reported to improve cognition, restored mitochondrial function and facilitates ATP synthesis (Beal, 2004; Turunen et al., 2004; Ishrat et al., 2006; Bentinger et al., 2010). It has been further reported that supplementation of CoQ10 increases endogenous CoQ10 content in rat brain (Lenaz et al., 1998; Rauscher et al., 2001; Somayajulu et al., 2005; Ishrat et al., 2006; Kim and Suh, 2009; Hargreaves, 2014). It also possesses membrane stabilizing properties and provides neuroprotection in various models of neurodegenerative disorders (Beal, 2004; Turunen et al., 2004; Ishrat et al., 2006; Bentinger et al., 2010; Yang et al., 2008, 2010).

Minocycline, a second-generation tetracycline antibiotic capable of inhibiting caspase-1 and -3 activities, release of apoptogenic factors from mitochondria, as well as caspase independent neuronal cell death pathways (Wang et al., 2003a; Choi et al., 2007). It also suppress microglial activated production of IL-1β, IL-6, TNF-α, and NGF (Lenaz et al., 1998; Wang et al., 2003a,b; Ryu et al., 2004; Seabrook et al., 2006; Maier et al., 2007; Kim and Suh, 2009). Besides, neuroprotective effects has been well documented in several neurological conditions (Seabrook et al., 2006; Kim and Suh, 2009). Being lipophilic in nature, it easily crosses the blood brain barrier (BBB), in order to produce a neuroprotective effect (Rauscher et al., 2001; Ryu et al., 2004).

The neuroprotective effect of both CoQ10 and minocycline have been well demonstrated in the various experimental models of neurodegenerative disorders. In the present study, we investigated whether administration of minocycline along with CoQ10 exerts enhanced beneficial effects in the Aβ (1-42) model of AD.

## Materials and Methods

### Animals

Adult male Sprague–Dawley rats (180–200 g) from Central Animal House of the Panjab University, Chandigarh, were used. Animals were acclimatized to laboratory conditions prior to experimentation at room temperature. The animals were kept under standard conditions of 12 h light and dark cycle with food and water *ad libitum*. All the experiments were carried out between 09:00 and 15:00 h. The protocol was approved by the Institutional Animal Ethics Committee (IAEC/411, 11/9/2013) and carried out in accordance with the guidelines of Committee for Control and Supervision of Experimentation on Animals (CPCSEAs), Government of India on animal experimentation. In our study, male animals were used because in female estrogen hormones have been found to interfere with the memory.

### Surgery and Intrahippocampal Administration of Aβ (1-42)

Surgery was performed with procedure as described by Prakash and Kumar (2014). Animals were anesthetized with thiopental sodium (45 mg/kg, i.p.), head was positioned in a stereotaxic apparatus and skull was exposed. A midline sagittal incision was made in the scalp and two holes were drilled in the skull. The stereotaxic coordinates for hippocampal region were 2.00 mm posterior to bregma, 1.5 mm lateral to the sagittal suture and 1.0 mm beneath the cortical surface set according to rat brain atlas (Paxinos and Watson, 1986). Through a skull hole, a 28-gage Hamilton micro syringe of 10 μl units and piston of the syringe was lowered manually into each hippocampal region. Rats were infused intrahippocampally (i.h.) with either artificial cerebrospinal fluid (ACSF; in mmol/L: 147 NaCl, 2.9 KCl, 1.6 MgCl_2_, 1.7 CaCl_2_, and 2.2 dextrose) or Aβ (1-42) (1 μg/μl) (CAS No. SCP0038) dissolved in ACSF according to the methods described by Christensen et al. (2008) and Zhong et al. (2009). Aβ (1-42) and ACSF was administered 4 μL/ site by using a Hamilton micro syringe positioned in the stereotaxic apparatus. The scalp was then closed with a suture. After surgery, all animals received gentamicin (5 mg/kg, i.p.) to prevent sepsis. In sham group, surgery was identical and animal received the same volume of ACSF instead of Aβ (1-42). Further, it was left in place for a period of 2 min following, i.h., administration of Aβ (1-42) to promote its diffusion from the micro syringe. Special care of the animals was taken during the post-operative period.

### Drugs and Treatment Schedule

**FIGURE 1:**
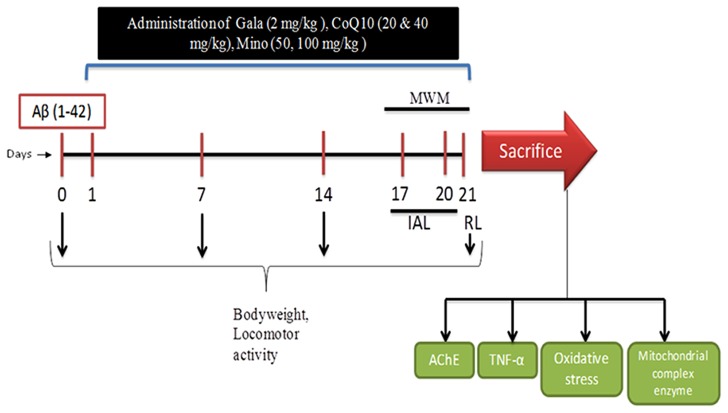
**Experimental Protocol (Aβ, amyloid beta; Gala, Galantamine; CoQ10, Coenzyme Q10; Mino, Minocycline; MWM, Morris water maze; IAL, initial acquired latency; RL, retention latency; AChE, acetylcholinesterase; TNF, Tumor Necrosis Factor)**.

Aβ (1-42) and Galantamine (Gala) were purchased from Sigma Chemicals, Pvt. Ltd., India. Coenzyme Q10 (CoQ10) was purchased from Himedia Laboratories, Pvt. Ltd., India and Minocycline was purchased from Ranbaxy Laboratories, Pvt., India. For drug administration Gala, CoQ10 and minocycline were suspended in 0.25% w/v sodium-carboxy-methyl-cellulose (Na-CMC) and administered in the dose of 5 ml/kg body weight for 21 days. Animals were selected randomly and divided on the basis of their body weights into several groups (*n* = 12 animals in each group) shown in **Table 1**. Doses of CoQ10 and minocycline were selected from the previous studies (Rauscher et al., 2001; Kalonia et al., 2012; Prakash and Kumar, 2014). Study was performed as shown in experimental protocol (**Figure 1**).

**Table 1:** Treatment groups.

S. No.	Group name	Treatment (mg/kg)
(1)	Naïve	Healthy animals
(2)	Sham	Surgery performed, ACSF administered (i.h.) (4 μl/site)
(3)	Aβ (1-42)	Single bilateral, i.h., Aβ (1-42) (1 μg/μl) [4 μl/site administered]
(4)	Gala (2)	Galantamine (2) administered in A (1-42) treated animals for 21 days
(5)	CoQ10 (20)	CoQ 10 (20) administered in Aβ (1-42) treated animals for 21 days
(6)	CoQ10 (40)	CoQ 10 (40) administered in Aβ (1-42) treated animals for 21 days
(7)	Mino (50)	Minocycline (50) administered in Aβ (1-42) treated animals for 21 days
(8)	Mino (100)	Minocycline (100) administered in Aβ (1-42) treated animals for 21 days
(9)	CoQ10 (20) + Mino (50)	CoQ 10 (20) + Minocycline (50) administered in Aβ (1-42) treated animals for 21 days
(10)	CoQ10 (40) + Mino (100)	CoQ 10 (40) + Minocycline (100) administered in Aβ (1-42) treated animals for 21 days


### Behavioral Parameters

#### Assessment of Cognitive Performance (Morris Water Maze Task)

Individual animals were tested in a spatial version of the Morris water maze (MWM) test (Morris, 1984; Kumar et al., 2007). The apparatus consisted of a circular water tank (150 cm diameter, 40 cm high, filled to a depth of 30 cm with water at 28 ± 1°C). A platform (4.5 cm diameter) was placed in one quadrant of the pool, 1 cm above the water level during the acquisition phase. The same platform was placed 1 cm below the water level for retention phase. The tank was located in a large room where there were several brightly colored clues external to the maze; these were visible from the pool and could be used by the rats for spatial orientation. During each trial, the position of the clues remained unchanged throughout the study and animal was subjected to four consecutive trials with a gap of 5 min. The animal was gently placed in the water of the pool between quadrants, facing the wall of the pool which was changed with each trial, and allowed 120 s to locate the platform. Then, animals were guided to remain on the platform for 20 s. If animal failed to reach the platform within 120 s, the same was guided to reach the platform and remained there for next 20 s.

##### Acquisition phase (training)

Animals received daily four consecutive training sessions from day 17 to 20. During the acquisition phase, each rat was put into the water in any one of four starting positions, the sequence of which was selected randomly. The time latency to reach the visual platform for escaping from water known as initial acquisition latency (IAL) also known as escape latency time (ELT) was measured.

##### Retention phase

After 24 h, i.e., on day 21 after IAL, the animal was released randomly at one of the edges facing the wall of the pool to assess for memory retention. Time taken by animal to find the hidden platform on day 21 following the start of Aβ (1-42) administration was recorded and termed as retention latency (RL) and time spent in the target quadrant (TSTQ) was also recorded.

#### Assessment of Locomotor Activity/Closed Field Activity

Locomotor activity was measured to rule out the interference of change in locomotor activity in the parameters of learning and memory. Each animal was placed in a square (30 cm) closed arena equipped with infra-red light sensitive photocells (digital actophotometer, IMCORP, India) and values expressed as counts per 5 min (Galpern and Cudkowicz, 2007; Kumar et al., 2007).

### Biochemical Analysis

The first set of animals (*n* = 5) were used for biochemical estimations including TNF-α levels. Biochemical tests were conducted after last behavioral test. Animals were killed by decapitation and their brains were taken out quickly to dissect hippocampus and cerebral cortex (Prakash and Kumar, 2014). The dissected brain parts were harvested and rinsed with ice-cold isotonic saline. Brains were then homogenized with ice-cold 0.1 mmol/l phosphate buffer (*p*H 7.4). The homogenate (10% w/v) was then centrifuged at 10,000 g for 15 min and the supernatant so formed was used for the biochemical estimations.

#### Measurement of Lipid Peroxidation

The extent of lipid peroxidation (LPO) in the hippocampus and cortex was determined quantitatively. The thiobarbituric acid reactive substances (TBARS) were measured at 532 nm using Perkin Elmer Lambda 20 Spectrophotometer (Norwalk, CT, USA) as per the method described by Wills (1966). The values were calculated using the molar extinction co-efficient of chromophore (1.56 × 105 (mol/l)^-1^ cm^-1^).

#### Estimation of Reduced Glutathione Levels

Reduced glutathione (GSH) was estimated according to the method as described by Ellman (1959). The yellow color developed was measured at 412 nm using Perkin Elmer Lambda 20 spectrophotometer. The results were calculated using molar extinction co-efficient of the chromophore (1.36 × 104 (mol/l)^-1^ cm^-1^).

#### Estimation of Nitrite

The accumulation of nitrite in the supernatant, an indicator of the production of nitric oxide (NO) was determined by a colorimetric assay with Greiss reagent (Green et al., 1982). The absorbance was measured at 540 nm using Perkin Elmer Lambda 20 spectrophotometer. The concentration of nitrite in the supernatant was determined from sodium nitrite standard curve.

#### Superoxide Dismutase Activity

Superoxide dismutase (SOD) activity was assayed by the method of Kono (1978). The change in absorbance was recorded for 2 min at 30 s intervals by measuring absorbance at 560 nm using Perkin Elmer Lambda 20 spectrophotometer.

#### Catalase Activity

Catalase activity was assessed by the method of Luck (1965). The change in absorbance was recorded for 2 min at 30 s interval at 240 nm using Perkin Elmer Lambda 20 spectrophotometer. The results were expressed as micromoles of hydrogen peroxide decomposed/min/mg of protein.

#### Acetylcholinesterase (AChE) Activity

The AChE activity was assessed by Ellman et al. (1961). The change in absorbance was measured for 2 min at 30 s interval at 412 nm using the Perkin-Elmer Lambda 20 spectrophotometer. Results were expressed as micromoles of acetylthiocholine iodide hydrolyzed/min/mg of protein.

#### Protein Content Estimation

The protein content was estimated by Biuret (Gornall et al., 1949) using bovine serum albumin as a standard.

### Mitochondrial Complex Estimation

#### Isolation of Rat Brain (Cortex and Hippocampus) Mitochondria for Enzyme Complex Assay

Second set of animals (*n* = 5) was used for mitochondrial complex estimation. The cortex and hippocampus was homogenized in an isolation buffer with EGTA (215 mM Mannitol, 75 mM sucrose, 0.1% BSA, 20 mM HEPES, 1 mM EGTA, *p*H-7.2). Homogenates were centrifuged at 13,000 *g* for 5 min at 4°C. Pellets were resuspended in isolation buffer with EGTA and spun again at 13,000 *g* for 5 min. The resulting supernatants were transferred to new tubes and topped off with isolation buffer with EGTA and again spun at 13,000*g* for 10 min. Pellets containing pure mitochondria were resuspended in isolation buffer without EGTA (Berman and Hastings, 1999).

#### Nicotinamide Adenine Dinucleotide (NADH) Dehydrogenase Activity

Complex-I was measured spectrophotometrically by the method of King and Howard (1967). The method involves catalytic oxidation of NADH to NAD^+^ with subsequent reduction in cytochrome C. The reaction was initiated by addition of requisite amount of solubilized mitochondrial sample and followed by an absorbance change at 550 nm for 2 min.

#### Succinate Dehydrogenase (SDH) Activity

Succinate Dehydrogenase (SDH) was measured spectrophotometrically according to King (1967). The method involves oxidation of succinate by an artificial electron acceptor, potassium ferricyanide. The reaction was initiated by the addition of mitochondrial sample and absorbance change was followed at 420 nm for 2 min.

#### 3-(4,5-dimethylthiazol-2-yl)-2,5-diphenyltetrazolium (MTT) Assay

Also known as mitochondrial redox activity assay, this method is based on the *in vitro* studies to evaluate mitochondrial redox activity through the conversion of MTT tetrazolium salt to formazan crystals by mitochondrial respiratory chain reactions in isolated mitochondria by the method of Liu et al. (1997). The absorbance of the resulting medium was measured by an ELISA reader at 580 nm wavelength.

#### Cytochrome Oxidase Activity

Cytochrome oxidase activity was assayed in brain mitochondria according to the method of Sotocassa et al. (1967). The reaction was started by the addition of solubilized mitochondrial sample and absorbance change was recorded at 550 nm for 2 min.

### Estimation of TNF-α Activity

The same first set of animals was used for the estimation of TNF-α level in rat brain. The quantification of cytokine TNF-α was performed as per the instruction of R&D Systems Quantikine rat TNF-α immunoassay kit (R&D Systems, Minneapolis, MN, USA). The Quantikine rat TNF-α immunoassays is 4.5 h solid phase ELISA designed to measure rat TNF-α level. Briefly, the assays employ the sandwich enzyme immunoassay technique (Prakash and Kumar, 2014). Monoclonal antibody specific for rat TNF-α have been pre-coated in the micro plate. Standards, control, and samples are pipette into the wells and any rat-TNF-α present is bound by the immobilized antibody. After washing away any unbound substance an enzyme linked polyclonal antibody specific for rat TNF-α was added to the wells. Following a wash to remove any unbound antibody-enzyme reagent, a substrate solution is added to the wells. The enzyme reaction yields a blue product that turns yellow when the stop solution is added. The intensity of the color measured is in proportion to the amount of rat TNF-α bound in the initial steps. The sample values are then read off the standard curve.

### Histopathological Analysis by Hematoxylin and Eosin Staining (H and E Staining)

The remaining third set of animals was used for histopathological analysis. Animals were sacrificed by decapitation immediately after the last behavioral test (Prakash and Kumar, 2014). The brains were removed and transferred to formalin (10% v/v). The brain tissues were embedded in paraffin blocks and sectioned into 3 mm thickness with the help of a microtome. The brain sections (5–10 μm) thick were de-waxed and stained with hematoxylin and eosin. Briefly, sections were immersed in the filtered hematoxylin solution for 1 min followed by rinsing with tap water. Then, the sections were immersed in eosin stain for 1–2 min and rinsed thoroughly with tap water followed by dehydration in ascending alcohol solutions (50, 70, 80, 95, and 100%). Now the sections were mounted on labeled slides. The stained sections were viewed under a binocular microscope (Nikon Eclipse 80i, Nikon Instruments Inc., USA) at 40× for evaluating the hippocampal regions of the brain for morphological changes and photographed (Galpern and Cudkowicz, 2007). The particular hippocampal areas were analyzed for the presence and density of pyknotic nuclei. The number of such pynotic nuclei per square pixel were analyzed using computer based image analysis (Image J 1.42q, NIH, USA).

### Statistical Analysis

Graph Pad Prism (Graph Pad Software, San Diego, CA, USA) was used for all statistical analysis. Values are expressed as mean ± SEM. The behavioral assessment data were analyzed by a repeated measures two-way analysis of variance (ANOVA) with drug-treated groups as between sessions and as within-subjects factors. The biochemical estimations were analyzed by one-way ANOVA. *Post hoc* comparisons between groups were made using Tukey’s test. The *P* < 0.05 was considered significant.

## Results

### Effect of CoQ10, Minocycline and Their Combinations on Spatial Navigation Task on Morris Water Maze in Aβ (1-42) Treated Animals

**FIGURE 2:**
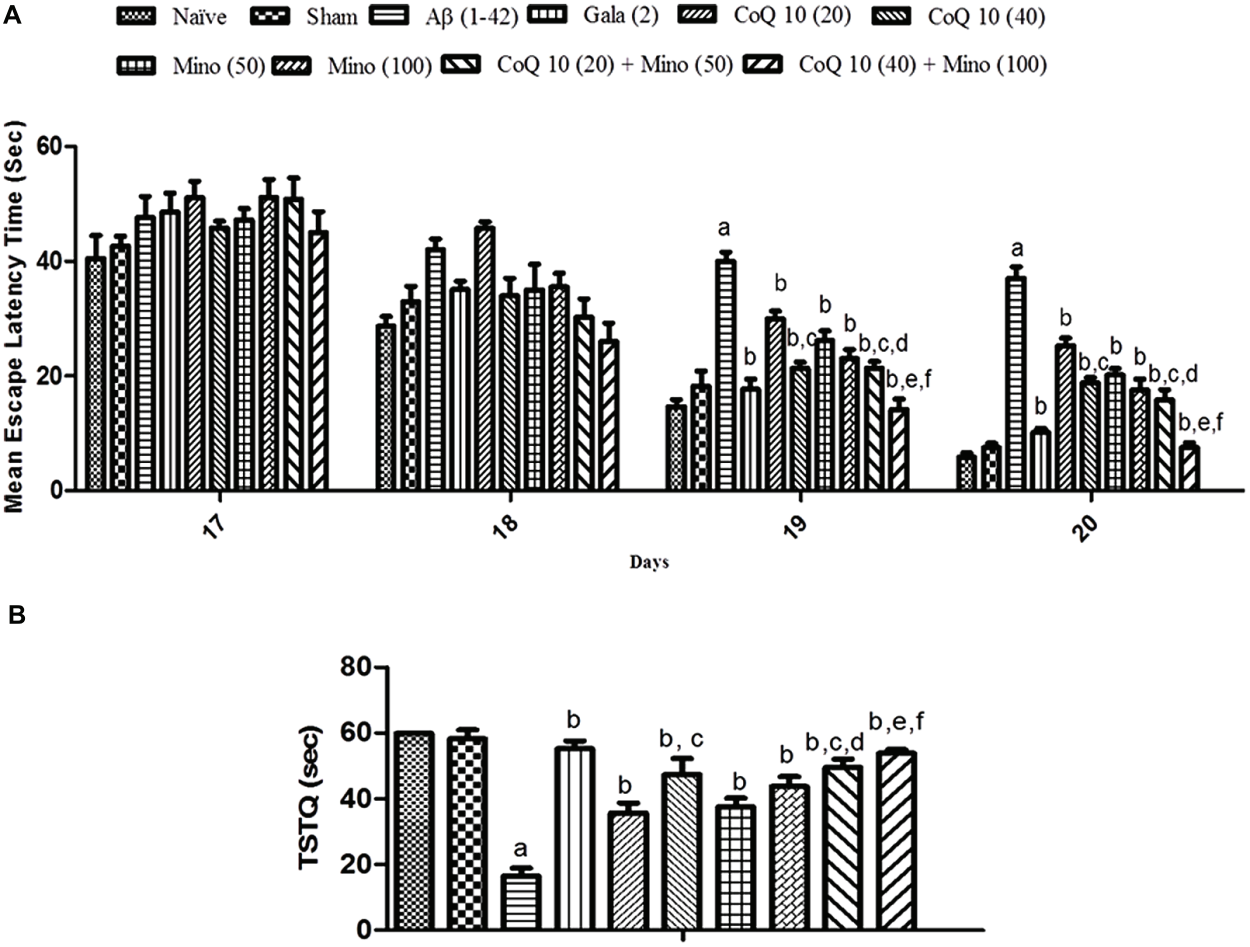
**(A)** Effect of CoQ10 and minocycline alone and in combination on memory performance in Morris water maze acquisition phase in Aβ (1-42) treated rats. Data expressed as mean ± SEM ^a^*P* < 0.05 compared to sham group, ^b^*P* < 0.05 compared to Aβ (1-42) treated group, ^c^*P* < 0.05 compared to CoQ10 (20) group, ^d^*P* < 0.05 compared to Mino (50) group, ^e^*P* < 0.05 compared to CoQ10 (40); ^f^*P* < 0.05 compared to Mino (100) group (Two way ANOVA followed by Bonferroni’s *t*-test). **(B)** Effect of CoQ10 and minocycline alone and in combination on time spent in the target quadrant (TSTQ) in the Morris maze retention phase (21st day) in Aβ (1-42) treated rats. Data expressed as mean ± SEM ^a^*P* < 0.05 compared to sham group, ^b^*P* < 0.05 compared to Aβ (1-42) treated group, ^c^*P* < 0.05 compared to CoQ10 (20) group, ^d^*P* < 0.05 compared to Mino (50) group, ^e^*P* < 0.05 compared to CoQ10 (40), ^f^*P* < 0.05 compared to Mino (100) group (One way ANOVA followed by Tukey’s test). (Aβ, Amyloid beta; Gala, Galantamine; CoQ10, Coenzyme Q 10; Mino, Minocycline).

Sham treated animals did not produce any significant effect on memory performance in MWM test as compared to a naïve group of animals. However, Aβ (1-42) treatment significantly caused a downward trend in ELT on subsequent water-maze exposure during training session (from day 17 to 20) as compared to the sham group indicating poor learning performance. Chronic treatment with CoQ10 (20 and 40 mg/kg), and minocycline (50 and 100 mg/kg) significantly (*P* < 0.05) improved memory performance and shortened escape latency as compared to Aβ (1-42) treated group. Further, treatment of CoQ10 (20 and 40 mg/kg) in combination with minocycline (50 and 100 mg/kg) significantly improved cognitive performance as compared to their effect *per se* in Aβ (1-42) treated animals. Their protective effect was comparable to that of Gala (2 mg/kg) treatment in Aβ (1-42) treated animals (**Figure 2A**).

Sham treatment did not produce any significant effect on the time spent in the target quadrant (TSTQ) as compared to naïve animals. Aβ (1-42) treated animals performed poorly and failed to recollect the location of the submerged platform on the 21st day, thus spending significantly less time in the target quadrant as compared to the sham group. However, CoQ10 (20 and 40 mg/kg), and minocycline (50 and 100 mg/kg) treatment for 21 days significantly increased TSTQ as compared to Aβ (1-42) treated group. Further, treatment of CoQ10 (20 and 40 mg/kg) in combination with minocycline (50 and 100 mg/kg) significantly (*p* < 0.05) improved cognitive performance (increased TSTQ) as compared to their effect *per se* in Aβ (1-42) treated animals. Their protective effect was comparable to that of Gala (2 mg/kg) treatment in Aβ (1-42) treated animals (**Figure 2B**).

### Effect of CoQ10, Minocycline and Their Combinations on Locomotor Activity in Aβ (1-42) Treated Animals

**FIGURE 3:**
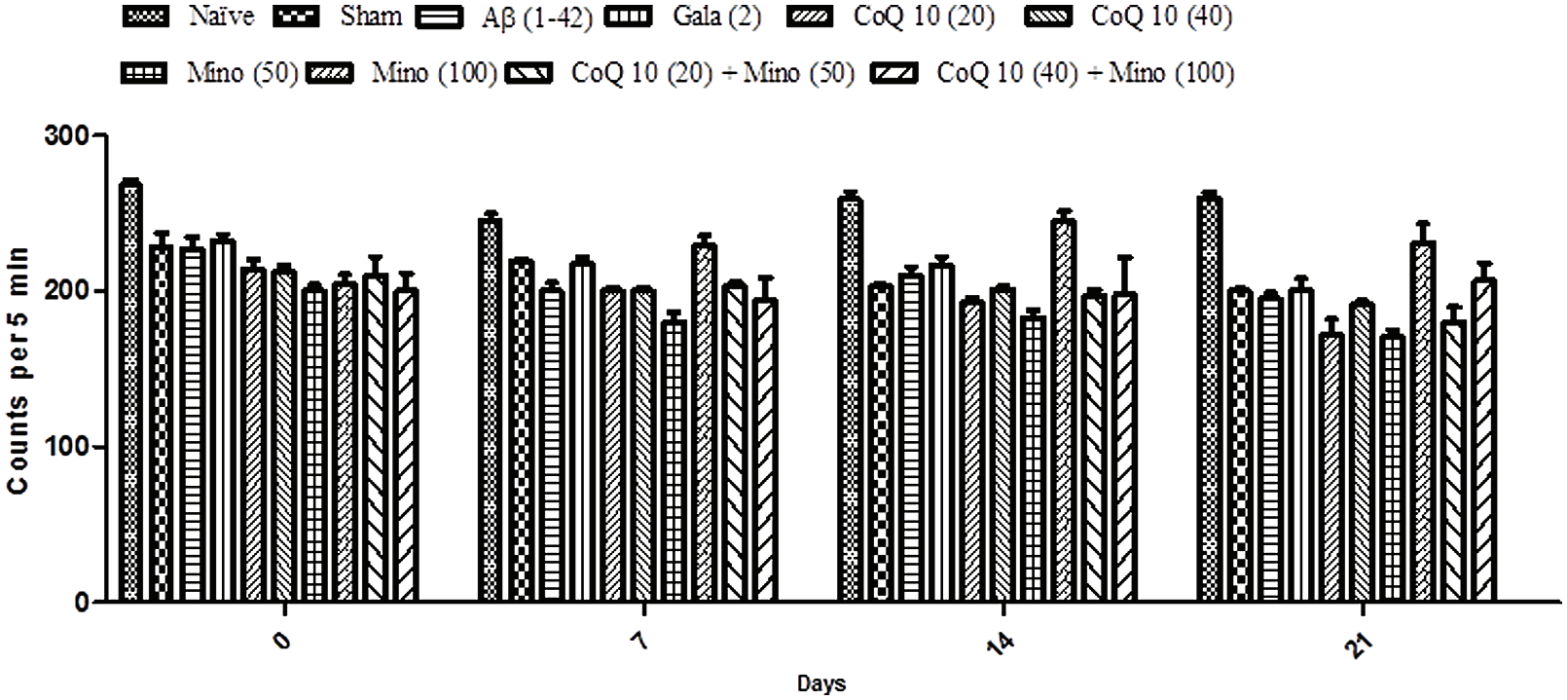
**Effect of CoQ10 and minocycline alone and in combination on locomotor activity (ambulation) in Aβ (1-42) treated rats.** Data expressed as mean ± SEM (Two way ANOVA followed by Bonferroni’s test). (Aβ (1-42), Amyloid beta; Gala, Galantamine; CoQ10, Coenzyme Q 10; Mino, Minocycline).

Locomotor activity in all groups was almost same before the start of surgery. However, there is no significant effect in mean scores of locomotor activity between naïve, sham treated and Aβ (1-42) treated animals. Chronic administration of CoQ10 (20 and 40 mg/kg) and minocycline (50 and 100 mg/kg) did not produce any significant effect on the locomotor activity as compared to Aβ (1-42) treated group. Further, combination of CoQ10 (20 and 40 mg/kg) with minocycline (50 and 100 mg/kg) in Aβ (1-42) treated animals did not produce any effect on locomotor activity on 7th, 14th, and 21st day as compared to Aβ (1-42) treated animals (**Figure 3**).

### Effect of CoQ10, Minocycline and Their Combinations on Oxidative Stress Parameters in Aβ (1-42) Treated Animals

Single bilateral, i.h., Aβ (1-42) administration caused significant rise in LPO, nitrite concentration and depletion of reduced GSH, SOD and catalase levels in hippocampus and cortex as compared to sham group. However, chronic CoQ10 (20 and 40 mg/kg) and minocycline (50 and 100 mg/kg) administration significantly (*p* < 0.05) attenuated oxidative stress (reduced the elevated MDA, nitrite concentration and restored SOD, catalase and GSH levels), as compared to Aβ (1-42) treated animals (*P* < 0.01). Further, treatment of CoQ10 (20 and 40 mg/kg) in combination with minocycline (50 and 100 mg/kg) significantly (0.05) potentiated their protective effect as compared to their effects alone in Aβ (1-42) treated animals. Eventually, their protective effect was comparable to that of Gala (2 mg/kg) treatment in Aβ (1-42) treated animals (*P* < 0.05) (**Table 2**).

**Table 2:** Effect of CoQ10 and minocycline on oxidative stress parameters in Aβ (1-42) treated animals.

Treatment group (mg/kg)	Brain Region	LPO (nmol MDA/mg of protein) (% of Sham)	Nitrite (μmol/mg of protein) (% of Sham)	GSH (nmol of GSH/mg Pr) (% of Sham)	Catalase (μM of H_2_O_2_/min/mgpr) (% of Sham)	SOD (Units/mg of protein) (% of Sham)
Naïve	Cortex Hippocampus	0.0184 ± 0.6 (97) 0.0246 ± 0.3 (95)	239.6 ± 2.1 (101) 181.8 ± 4.5 (98)	0.61 ± 0.02 (105.1) 0.69 ± 0.08 (97.1)	7.36 ± 0.52 (105.2) 6.22 ± 0.08 (101)	0.49 ± 0.017 (102.8) 0.593 ± 0.02 (106.0)
						


Sham (ACSF)	Cortex Hippocampus	0.019 ± 0.88 (100) 0.0259 ± 0.01(100)	237.3 ± 1.4 (100) 285.6 ± 2.18 (100)	0.58 ± 0.02 (100) 0.71 ± 0.03 (100)	7 ± 0.25 (100) 6.16 ± 0.32 (100)	0.48 ± 0.04 (100) 0.56 ± 0.02 (100)
						


Aβ (1-42) (1 μg/μl)	Cortex Hippocampus	0.0912 ± 0.3^a^ (480) 0.134 ± 0.25^a^ (520)	643.08 ± 2.09^a^ (341.4) 828.2 ± 2.60^a^ (290.7)	0.239 ± 0.21^a^ (41.3) 0.238 ± 0.08^a^ (33.6)	2 ± 0.05^a^ (28.5) 2.20 ± 0.12^a^ (35.8)	0.103 ± 0.0012^a^ (21.5) 0.164 ± 0.88^a^ (29.4)
						


Gala (2)	Cortex Hippocampus	0.0221 ± 0.32^b^ (116.8) 0.0318 ± 0.65^b^ (122.8)	285.5 ± 7.5^b^ (119.9) 295.8 ± 2.84^b^ (103.6)	0.49 ± 0.02^b^ (89) 0.673 ± 0.03^b^ (94.9)	6.559 ± 0.24^b^ (93.7) 5.69 ± 0.15^b^ (92.5)	0.449 ± 0.23^b^ (93.7) 0.505 ± 0.21^b^ (90.3)
						


CoQ 10 (20)	Cortex Hippocampus	0.0699 ± 0.1^b^ (368.42) 0.107 ± 0.13^b^ (416.77)	611.7 ± 1.64^b^ (257.8) 760.8 ± 6.42^b^ (266.4)	0.103 ± 0.01^b^ (47.7) 0.096 ± 0.21^b^ (43.5)	2.73 ± 0.16^b^ (39 2.5 ± 0.17^b^ (40.5)	0.172 ± 0.2^b^ (36.04) 0.239 ± 0.02 ^b^ (42.8)
						


CoQ 10 (40)	Cortex Hippocampus	0.028 ± 0.25^b,c^ (152.6) 0.053 ± 0.1^b,c^ (208)	447.7 ± 6.17^b,c^ (188.7) 430.1 ± 4.17^b,c^ (150.6)	0.406 ± 0.18^b,c^ (70) 0.503 ± 0.03^b,c^ (70.8)	3.31 ± 0.10^b,c^ (47.4) 4.27 ± 0.04^b,c^ (69.4)	0.201 ± 0.11^b,c^ (42) 0.305 ± 0.28^b,c^ (54.6)
						


Mino (50)	Cortex Hippocampus	0.0589 ± 0.31^b^ (310) 0.086 ± 0.13^b^ (333.9)	333.3 ± 9.27^b^ (240.4) 432.6 ± 12.8^b^ (249)	0.346 ± 0.34 ^b^ (59.8) 0.440 ± 0.52^b^ (62.1)	2.205 ± 0.38^b^ (31.5) 3.22 ± 0.46^b^ (52.43)	0.109 ± 0.04^b^ (22.9) 0.149 ± 0.26 ^b^ (26.7)
						


Mino (100)	Cortex Hippocampus	0.0329 ± 0.16^b^ (173.6) 0.0482 ± 0.9^b^ (186.4)	451.5 ± 4.16^b^ (190.3) 497.2 ± 18.5^b^ (174.1)	0.428 ± 0.30^b^ (73.9) 0.492 ± 0.25^b^ (69.3)	3.36 ± 0.41^b^ (48.1) 3.82 ± 0.52^b^ (62.1)	0.209 ± 0.6^b^ (43.7) 0.359 ± 0.45^b^ (64.2)
						


CoQ10 (20) + Mino (50)	Cortex Hippocampus	0.0401 ± 0.5^b,c,d^ (211.5) 0.0699 ± 0.2^b,c,d^ (270.2)	463.4 ± 2.9^b,c,d^ (195.3) 621.7 ± 1.9^b,c,d^ (217.7)	0.454 ± 0.17^b,c,d^ (78.3) 0.511 ± 0.2^b,c,d^ (72.1)	3.5 ± 0.3^b,c,d^ (50) 4.8 ± 0.23^b,c,d^ (77.9)	0.289 ± 0.3^b,c,d^ (60.4) 0.409 ± 0.5^b,c,d^ (73.2)
						


CoQ10 (40) + Mino(100)	Cortex Hippocampus	0.0230 ± 0.8^b,e,f^(121.5) 0.0372 ± 0.23^b,e,f^ (144)	305.8 ± 12.4^b,e,f^ (128.9) 378.9 ± 3.17^b,e,f^ (132.7)	0.498 ± 0.9^b,e,f^ (86) 0.646 ± 0.3^b,e,f^ (91)	6.02 ± 0.84^b,e,f^ (86.1) 5.62 ± 0.15^b,e,f^ (91.3)	0.42 ± 0.40^b,e,f^ (88.6) 0.49 ± 0.15^b,e,f^ (87.7)


*Values are mean ± SEM.*

*^a^*P* < 0.05 compared to sham group, ^b^*P* < 0.05 compared to Aβ (1-42) treated group, ^c^*P* < 0.05 compared to CoQ10 (20) group, ^d^*P* < 0.05 compared to Mino (50) group,^e^*P* < 0.05 compared to CoQ10 (40), ^f^*P* < 0.05 compared to Mino (100) group (One way ANOVA followed by Tukey’s test). (Aβ, Amyloid beta; Gala, Galantamine; CoQ10, Coenzyme Q 10; Mino, Minocycline).*

### Effect of CoQ10, Minocycline and Their Combinations on Mitochondrial Enzyme Complex in Aβ (1-42) Treated Animals

**FIGURE 4:**
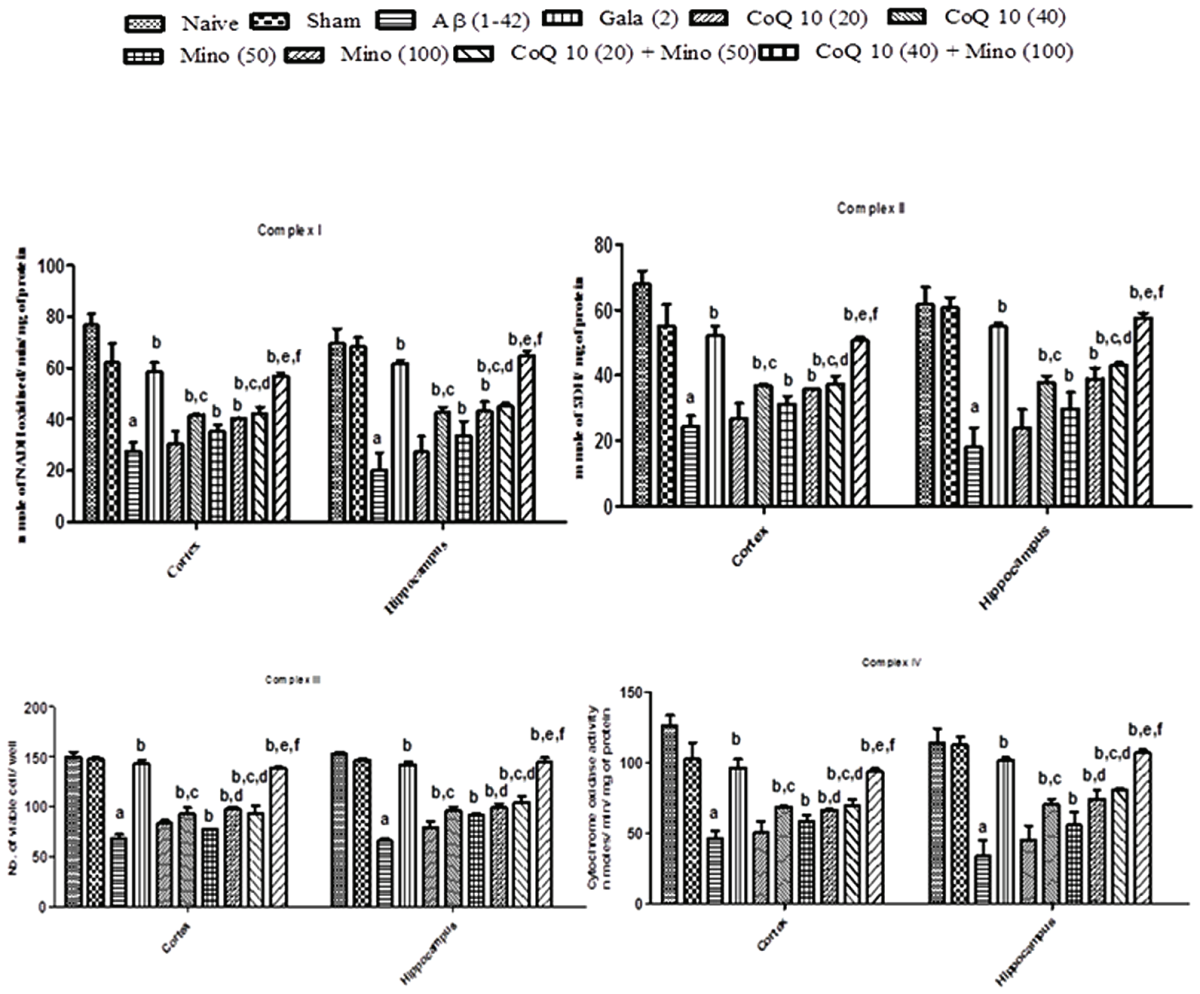
**Effect of CoQ10 and minocycline alone and in combination on mitochondrial respiratory enzymes in the hippocampus and cortex region of Aβ (1-42) treated rats.** Data expressed as mean ± SEM ^a^*P* < 0.05 compared to sham group; ^b^*P* < 0.05 compared to Aβ (1-42) treated group; ^c^*P* < 0.05 compared to CoQ10 (20) group; ^d^*P* < 0.05 compared to Mino (50) group; ^e^*P* < 0.05 compared to CoQ10 (40); ^f^*P* < 0.05 compared to Mino (100) group (One way ANOVA followed by Tukey’s test). (Aβ, Amyloid beta; Gala, Galantamine; CoQ10, Coenzyme Q 10; Mino, Minocycline).

Mitochondrial enzyme complex (NADH dehydrogenase, succinate dehydrogenase and cytochrome oxidase and cell viabilities activities) significantly depleted in hippocampus and cortex regions of Aβ (1-42) treated group as compared to sham group (*P* < 0.05). However, chronic CoQ10 (20 and 40 mg/kg) and minocycline (50 and 100 mg/kg) treatment significantly restored mitochondrial enzymes complex activities and increased cell viability as compared to Aβ (1-42) treated animals (*P* < 0.05). Further, treatment of CoQ10 (20 and 40 mg/kg) in combination with minocycline (50 and 100 mg/kg) significantly (*P* < 0.05) potentiated their protective effect which was significant (*P* < 0.05) in comparison with their effects alone in Aβ (1-42) treated animals. As expected, their protective effect was comparable to that of Gala (2 mg/kg) treatment in Aβ (1-42) treated animals (*P* < 0.05) (**Figure 4**).

### Effect of CoQ10, Minocycline and Their Combinations on AChE Activity in Aβ (1-42) Treated Animals

**FIGURE 5:**
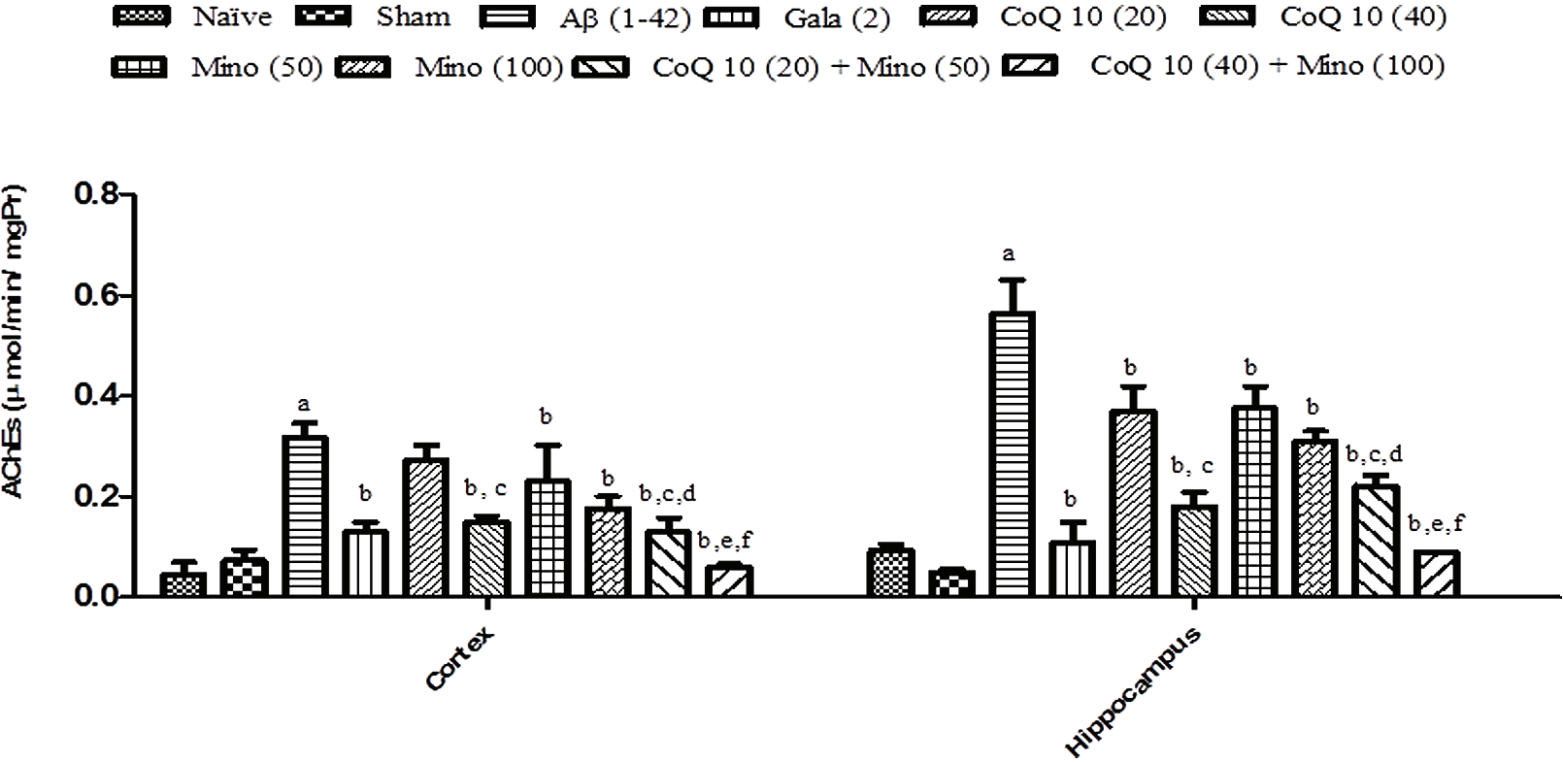
**Effect of CoQ10 and minocycline alone and in combination on AChE activity in the hippocampus and cortex region of Aβ (1-42) treated rats.** Data expressed as mean ± SEM ^a^*P* < 0.05 compared to sham group; ^b^*P* < 0.05 compared to Aβ (1-42) treated group; ^c^*P* < 0.05 compared to CoQ10 (20) group; ^d^*P* < 0.05 compared to Mino (50) group; ^e^*P* < 0.05 compared to CoQ10 (40); ^f^*P* < 0.05 compared to Mino (100) group (One way ANOVA followed by Tukey’s test). (Aβ, Amyloid beta; Gala, Galantamine; CoQ10, Coenzyme Q 10; Mino, Minocycline; AChE, acetylcholinesterase).

Single bilateral, i.h, Aβ (1-42) treatment significantly increased AChE activity in hippocampus and cortex regions as compared to the sham group (*P* < 0.05). However, treatment with CoQ10 (20 and 40 mg/kg) and minocycline (50 and 100 mg/kg) for 21 days significantly (*P* < 0.05) attenuated AChE activity which was significant as compared to Aβ (1-42) treated animals. Further, treatment of CoQ10 (20 and 40 mg/kg) in combination with minocycline (50 and 100 mg/kg) for 21 days significantly (*P* < 0.05) potentiate their protective effect in Aβ (1-42) treated animals. Finally, their protective effect was comparable to that of Gala (2 mg/kg) treatment in Aβ (1-42) treated animals (**Figure 5**).

### Effect of CoQ10, Minocycline and Their Combinations on Brain Hippocampus (TNF-α) Level in Aβ (1-42) Treated Animals

**FIGURE 6:**
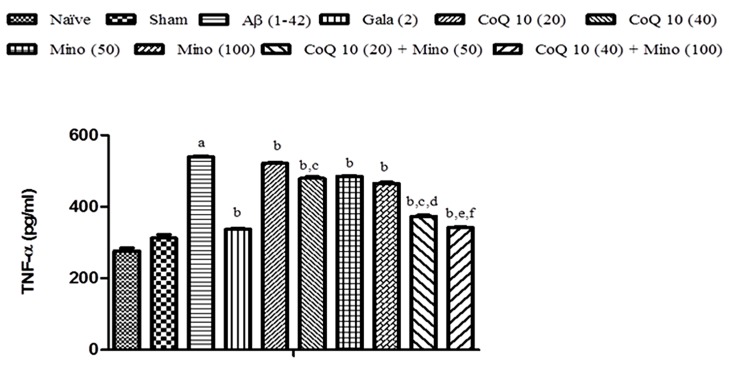
**Effect of CoQ10 and minocycline alone and in combination on TNF-α level in Aβ (1-42) treated rats.** Data expressed as mean ± SEM, ^a^*P* < 0.05 compared to sham group, ^b^*P* < 0.05 compared to Aβ (1-42) treated group, ^c^*P* < 0.05 compared to CoQ10 (20) group, ^d^*P* < 0.05 compared to Mino (50) group, ^e^*P* < 0.05 compared to CoQ10 (40), ^f^*P* < 0.05 compared to Mino (100) group (One way ANOVA followed by Tukey’s test). (Aβ, Amyloid beta; Gala, Galantamine; CoQ10, Coenzyme Q 10; Mino, Minocycline).

Single bilateral, i.h., Aβ (1-42) administration significantly elevated TNF-α level as compared to sham group. Chronic CoQ10 (20 and 40 mg/kg) and minocycline (50 and 100 mg/kg) administration significantly attenuated TNF-α level (*P* < 0.05) in the hippocampus region of Aβ (1-42) treated animals which was significant as compared to Aβ (1-42) treated group (*P* < 0.05). Further, treatment of CoQ10 (20 and 40 mg/kg) in combination with minocycline (50 and 100 mg/kg) significantly potentiated their protective effect in Aβ (1-42) treated animals. Their protective effect was comparable to that of Gala (2 mg/kg) treatment in Aβ (1-42) treated animals (**Figure 6**).

### Histopathological Examination

**FIGURE 7:**
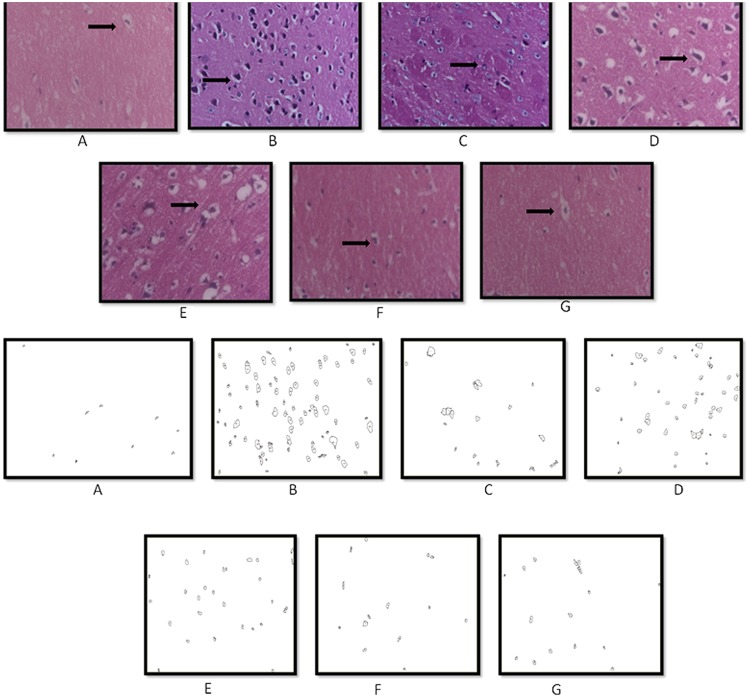
**Histopathological sections of rat brain stained with Hematoxylin and Eosin stain (H and E).** Representative photomicrographs of CA regions of hippocampus sections (magnification 10X, from **A–G**) of rat. **(A)** Sham: neurons are intact; **(B)** Aβ (1-42): inflammatory cells; **(C)** Gala (2): mild inflammation of neurons; **(D)** CoQ10 (20): moderate inflammation of neurons; **(E)** CoQ10 (40): moderate inflammation of neurons; **(F)** CoQ10 (20) + Minocycline (50): moderate inflammation of neurons; **(G)** CoQ10 (40) + Minocycline (100): mild inflammation of neurons (as shown by the arrow).

**FIGURE 8:**
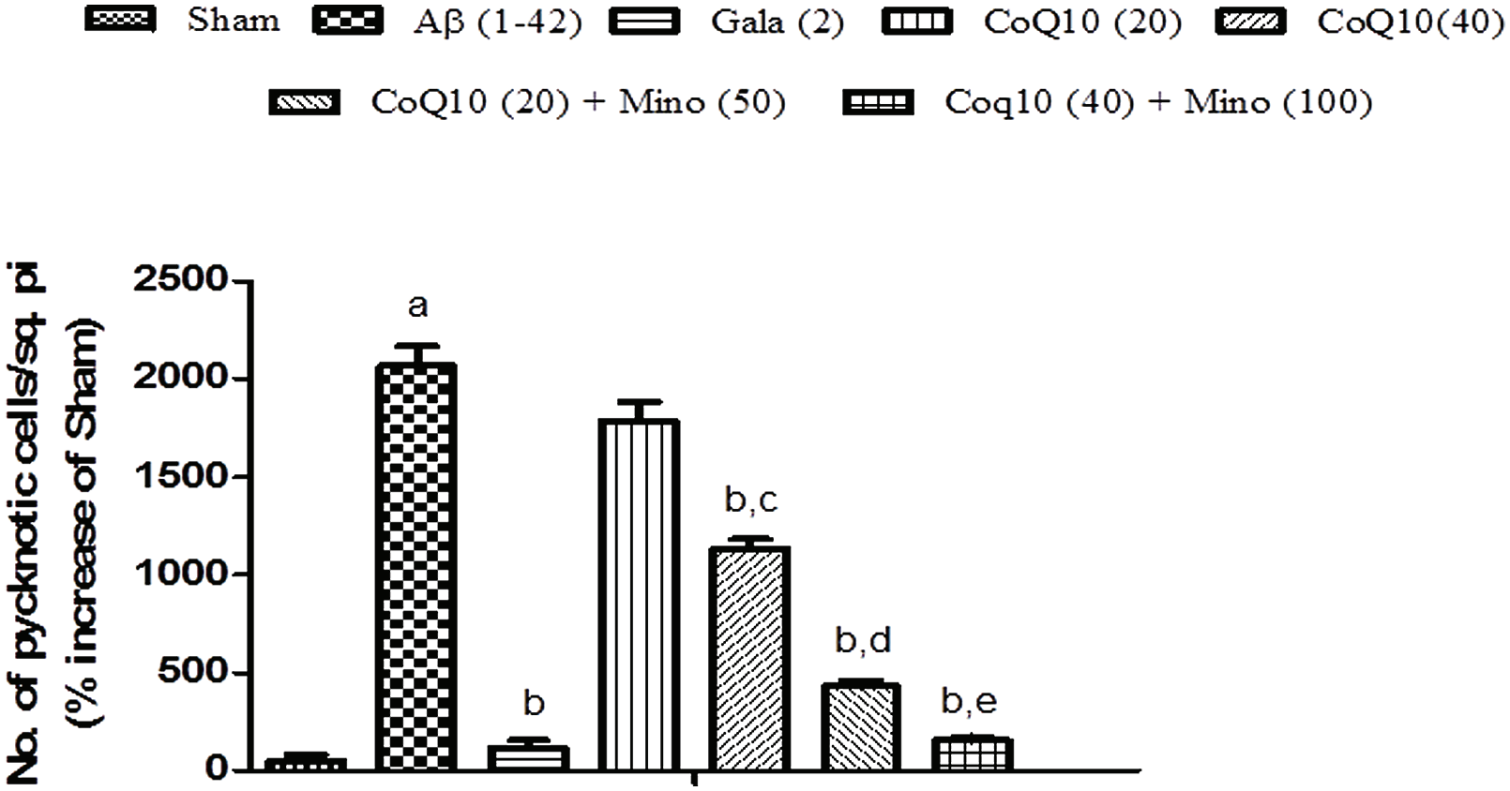
**Graph showing the effect of CoQ10 and Minocycline on pyknotic expressions per square pixel.** Data expressed as mean ± SEM, ^a^*P* < 0.05 compared to sham group, ^b^*P* < 0.05 compared to Aβ (1-42) treated group, ^c^*P* < 0.05 compared to CoQ10 (20) group, ^d^*P* < 0.05 compared to CoQ10 (20) group and Aβ (1-42) treated group, ^e^*P* < 0.05 compared to CoQ10 (40) and Aβ (1-42) treated group (One way ANOVA followed by Tukey’s test). (Aβ, Amyloid beta; Gala, Galantamine; CoQ10, Coenzyme Q 10; Mino, Minocycline).

Histopathological evaluation of brain tissue was carried on under light microscopy. Brain sections for the different groups of animal are shown in **Figure 7**. In the histopathological study, sham-treated brain showed undamaged neuronal cells as compared to naïve animal. However, disarrangement of various cell layers as well as the pyramidal neuronal cell loss and increase in the density of pyknotic cells (number of pyknotic cells, per 128800 square pixels of area; representing inflammation) were found in CA1 and CA3 hippocampal regions of brain in Aβ (1-42) treated animals which was significant as compared to sham group. At some places, cortical spongiosis was also observed in the brains of Aβ (1-42) treated animals. However, 21 days chronic CoQ10 (20 and 40 mg/kg) and minocycline (50 mg/kg) administration significantly attenuated the loss of neuronal cell density and reduction in the number of pyknotic cells as compared to Aβ (1-42) treated animals. Moreover, the neuroprotective effects of CoQ10 (20 and 40 mg/kg) were potentiated dose dependently by the combination of minocycline (50 and 100 mg/kg) where the integrity of the neuronal cell layer and reduced number of pyknotic cells displayed less neuroinflammation as compared to their effect alone. Their protective effect was comparable to that of Gala (2 mg/kg) treatment in Aβ (1-42) treated animals (*P* < 0.05) (**Figure 8**)

## Discussion

Alzheimer’s disease is a slow, progressive neurodegenerative problem with unknown etiology. It is a leading cause of senile dementia characterized by neuronal degeneration, cognitive dysfunction. Several mechanisms have been suggested to explain its complex pathophysiology particularly oxidative stress, neuroinflammation, mitochondrial dysfunction and apoptosis (Hampel et al., 2011; Braak and Del Tredici, 2012; Anand et al., 2014; Association, 2014; Kumar and Singh, 2015).

In our study, i.h., administration of Aβ (1-42) significantly impaired cognitive performance in MWM test as indicated by delayed transfer latency time to reach the platform (ELT) and shortened TSTQ as compared to sham group, suggesting that Aβ (1-42) induced impairment in spatial learning and memory. It also causes significant increase in AChE activity supporting an impairment of the cholinergic system, suggesting Aβ (1-42) causes cholinergic dysfunction. The findings of the present study revealed that, i.h., administration of Aβ (1-42) resulted in a significant increase in MDA; nitrite concentration, depleted reduced glutathione, SOD and catalase activities in Aβ (1-42) treated animals suggesting oxidative stress. The present study also indicated that, i.h., Aβ (1-42) accumulation significantly altered the activity of mitochondrial respiratory enzymes (NADH dehydrogenase, succinate dehydrogenase activity, cytochrome C oxidase) and impaired the neural cell viability of Aβ (1-42) treated animals as compared to sham treated animals suggesting mitochondrial dysfunction. In this study, i.h., administration of Aβ (1-42) significantly increased TNF-α level in Aβ (1-42) treated animals as compared to sham treated animals, indicating the role of neuroinflammation in Aβ (1-42) model of cognitive dysfunction.

Previous studies have also demonstrated the neuroprotective potential of both CoQ10 and minocycline in different neurological conditions (Rauscher et al., 2001; Beal, 2004; Ryu et al., 2004; Turunen et al., 2004; Somayajulu et al., 2005; Seabrook et al., 2006; Kim and Suh, 2009). However, their mechanism of action is not yet clear. Supporting to the above, the present study demonstrated the neuroprotective effect of CoQ10 and minocycline against Aβ (1-42) induced cognitive dysfunction. Therefore, beneficial treatment of CoQ10 along with minocycline in neurodegenerative disorders including AD with multiple pathogenic properties can be achieved through drugs targeting multiple pathways or polytherapeutic interventions directed toward specific aspect of the neurodegenerative phenotype (Kumar and Singh, 2015).

Coenzyme Q10 is a well known lipophilic antioxidant compound, has been reported to improve cognitive function, up regulates mitochondrial function and facilitates ATP synthesis (Ishrat et al., 2006; Hargreaves, 2014). Supplementation of CoQ10 increases brain endogenous Q10 content and affords as an antioxidant against free radical generation as well as oxidative modification of biomolecules (Ishrat et al., 2006; Hargreaves, 2014). It also provides significant improvement in patients with neurological disorders (Moreira et al., 2005; Ishrat et al., 2006; Stack et al., 2006; Hargreaves, 2014). The major role of CoQ is to transfer electrons between redox components of the ETC, to create a proton gradient across the inner mitochondrial membrane and drive ATP formation and as a powerful antioxidant that has been shown to be protective against oxidative stress (Moreira et al., 2005; Lenaz et al., 2007). Thus, CoQ10 protects *in vivo* against oxidation of lipids, proteins, and DNA. Previous evidence has suggested an intimate link between an excessive generation of ROS/RONS and development of hippocampal neuronal death (Ishrat et al., 2006; Galpern and Cudkowicz, 2007; Swomley et al., 2014). In the present study, chronic treatment with CoQ10 (20 and 40 mg/kg) in Aβ (1-42) treated animals significantly attenuated an impairment of spatial learning and memory task performance, AChE activities, mito-oxidative damage, restored mitochondrial respiratory enzyme complex activities and TNF-α level suggesting its anti-oxidant, mitochondrial restoring and anti-inflammatory action as compared to Aβ (1-42) treated animals.

Minocycline, a semi-synthetic second-generation tetracycline analog has both antimicrobial and anti-inflammatory activity (Kim and Suh, 2009). It also possesses efficacy against broad range of neurological disorders with good clinical safety record (Ryu et al., 2004; Seabrook et al., 2006; Maier et al., 2007; Kim and Suh, 2009). Previous studies on animal model (Smac/Diablo) have showed that minocycline prevented the release of cytochrome c in isolated mitochondria from, suggesting mitochondria as a direct target of minocycline (Wang et al., 2003c). Minocycline, has also known to inhibit the formation of Aβ aggregates and disassembles the performed fibrils (Burgos-Ramos et al., 2009). In the present study, chronic treatment with minocycline improved spatial learning and memory task performance, reduced AChE activities, mito-oxidative damage, restored mitochondrial respiratory enzyme complex activities and attenuated TNF-α level, suggesting its anti-oxidant, mitochondrial restoring and anti-inflammatory action as compared to Aβ (1-42) treated animals.

Further, chronic treatment of CoQ10 in combination with minocycline animals significantly enhanced their neuroprotective effect (attenuated spatial learning and memory performance task, AChE activities, mito-oxidative damage, restored the mitochondrial respiratory enzyme activities and TNF-α level) suggesting their synergistic action. In the present study, Aβ (1-42) administration causes significant damage in the hippoacampal and cortical regions of the rat brain that plays important role in the learning and memory. For the histopathological evaluations of the rat brains, hippocampal region were targeted and it was observable that brain areas of Aβ (1-42) treated animals had significantly higher density of pyknotic neuronal cells suggesting degeneration and disrupted functioning of the hippocampal neurons. Besides, CoQ10 in combination with minocycline, significantly improved cell layer organization, reduced density of pyknotic cells and pyramidal cell in the CA1 and CA3 regions as compared to their effects alone in Aβ (1-42) treated rat brain hippocampus.

## Conclusion

**FIGURE 9:**
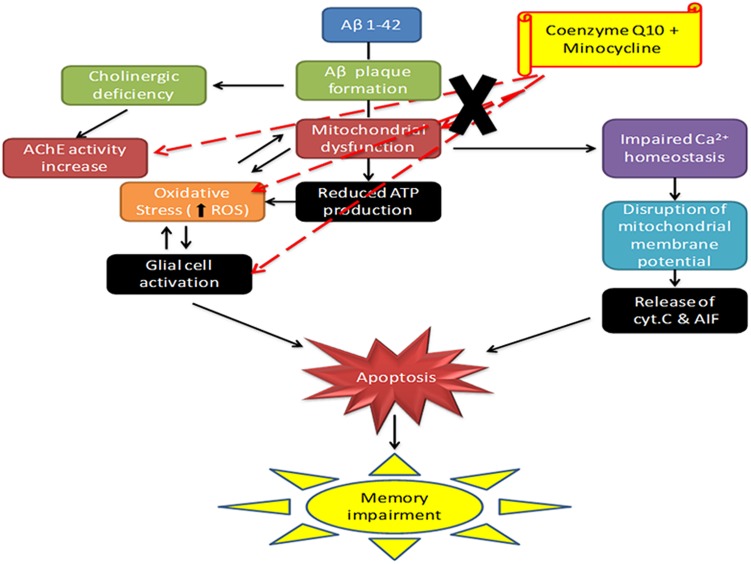
Schematic diagram showing the mechanism of action of CoQ10 and Minocycline (CoQ10, coenzyme Q10; Minocycline, minocycline; AChE, acetylcholinesterase; Cyt. C, cytochrome c).

In support, these results highlight anti-oxidant, anti-inflammatory and mitochondrial restoring property to CoQ10 and minocycline combination. Further, their combination targets at multiple sites in AD pathology as depicted in **Figure 9**. It seems that minocycline in combination with CoQ10 significantly modulate their neuroprotective effect against Aβ (1-42) induced cognitive dysfunction. Study further provides a hope that this drug combination could be used successfully in the management of AD.

## Author Contributions

AS performed the research and wrote the manuscript. Prof. AK analyzed and critically evaluate the manuscript.

## Conflict of Interest Statement

The authors declare that the research was conducted in the absence of any commercial or financial relationships that could be construed as a potential conflict of interest.
